# Invasive meningococcal disease in England: assessing disease burden through linkage of multiple national data sources

**DOI:** 10.1186/s12879-015-1247-7

**Published:** 2015-12-01

**Authors:** Shamez N Ladhani, Pauline A Waight, Sonia Ribeiro, Mary E Ramsay

**Affiliations:** Immunisation, Hepatitis and Blood Safety Department, Public Health England, 61 Colindale Avenue, London, NW9 5EQ UK

**Keywords:** Invasive meningococcal disease, Data linkage, Hospitalisation, Disease burden, Outcome

## Abstract

**Background:**

In England, Public Health England conducts enhanced surveillance of invasive meningococcal disease (IMD). The continuing decline in reported IMD cases has raised concerns that the MRU may be underestimating true IMD incidence.

**Methods:**

We linked five national datasets to estimate disease burden over five years, including PHE Meningococcal Reference Unit (MRU) confirmations, hospital episode statistics (HES), electronic reports of significant infections by National Health Service (NHS) Hospitals, death registrations and private laboratory reports.

**Results:**

During 2007–11, MRU confirmed 5115 IMD cases and 4275 (84 %) matched to HES, including 3935 (92 %) with A39* (meningococcal disease) and 340 (8 %) with G00* (bacterial meningo-encephalitis) ICD-10 codes. An additional 2792 hospitalised cases with an A39* code were identified in HES. Of these, 1465 (52 %) matched to one of 53,806 samples tested PCR-negative for IMD by MRU and only 73 of the remaining 1327 hospitalised A39* cases were confirmed locally or by a private laboratory. The characteristics of hospitalised cases without laboratory confirmation were similar to PCR-negative than PCR-positive IMD cases.

**Conclusions:**

Interrogation of multiple national data sources identified very few laboratory confirmations in addition to the MRU-confirmed cases. The large number of unconfirmed and PCR-negative cases in HES suggests increased awareness among clinicians with low thresholds for hospitalising patients with suspected IMD.

**Electronic supplementary material:**

The online version of this article (doi:10.1186/s12879-015-1247-7) contains supplementary material, which is available to authorized users.

## Background

Invasive meningococcal disease (IMD) is associated with significant case fatality and long-term morbidity despite availability of effective antibiotics and intensive care support in industrialised countries [1, 2]. Efforts to control the disease have, therefore, focussed on prevention, primarily through vaccination [3]. Most cases of IMD are caused by one of six meningococcal capsular groups, which are characterised by their unique polysaccharide capsule (A, B, C, W, X or Y) [4]. In the United Kingdom, meningococcal group C (MenC) is rare because of a successful national immunisation programme and group B (MenB) is responsible for most IMD cases across all age groups [5].

In England, national surveillance of laboratory-confirmed IMD is conducted by Public Health England (PHE), through the Meningococcal Reference Unit (MRU), which provides a national service for species confirmation, grouping, typing, subtyping and antimicrobial susceptibility testing of all invasive *Neisseria meningitidis* isolates. The MRU also provides free non-culture PCR confirmation of meningococcal diagnosis (including genogroup and genosubtype analysis) for clinical specimens that are routinely submitted by National Health Service (NHS) hospitals in England [5]. We have recently highlighted the added value of the PCR-testing service because of its high sensitivity and specificity, which has proved invaluable in achieving high case ascertainment rates for IMD surveillance [6]. However, whilst the current surveillance provides important information on the epidemiology of IMD and trends over time, it may underestimate the true burden of disease if clinical specimens are not submitted to the MRU or if hospitals submit their clinical isolates to private laboratories that provide a similar service. Cases that are diagnosed clinically without laboratory-confirmation, too, would not be included in the current surveillance. This may be more important for the less prevalent meningococcal capsular groups, which have more varied clinical presentations and often affect older adults with co-morbidities [7]. Likewise, meningitis cases may be missed if lumbar punctures are not performed [8].

In order to define the true burden of IMD, it is important to evaluate routine surveillance against alternate data sources. Accurate data form an essential component for modelling the potential impact (including cost-effectiveness) of any new vaccine which, in turn, can be used to inform national vaccination policy. The use of existing national datasets as a means of undertaking health research and improving public health monitoring offers exciting opportunities to undertake very large scale studies at very low cost. This study aimed to integrate epidemiological, clinical, microbiological and outcome data using multiple national datasets to assess the burden of IMD in England over a five-year period.

## Methods

PHE conducts enhanced surveillance of vaccine-preventable infections in England. For this study, five independent national datasets covering the five-year period during 2007–2011 were de-duplicated prior to linkage (Table 1). In addition to data collected through provision of a national reference laboratory service, PHE also routinely receives electronic notifications of clinically significant isolates by NHS laboratories through LabBase2 and reports of PCR-confirmed cases from private laboratories that support microbiology services for NHS hospitals. PHE also has access to Hospital Episode Statistics (HES), an online database containing electronic records of patients admitted to NHS hospitals, and receives annual electronic reports of all death registrations in England from the Office for National Statistics (ONS). PHE has been granted access to these data, through Regulation 3 of The Health Service (Control of Patient Information) Regulations 2002 (http://www.legislation.gov.uk/uksi/2002/1438/regulation/3/made) in order to fulfil its legal responsibility to monitor the impact, safety and effectiveness of the national immunisation programmes.

**Table 1:** Summary and characteristics of datasets used for the linkage study to estimate the total burden of invasive meningococcal disease (IMD) in England over a five-year period (2007–11)

PHE MRU laboratory-confirmed IMD cases	PHE MRU provides a national service for meningococcal species confirmation and molecular characterisation of invasive clinical isolates as well as a free national polymerase chain reaction (PCR) service to detect meningococcal DNA in clinical specimens from National Health Service (NHS) hospital laboratories throughout England. The MRU dataset maintains a record of all laboratory-confirmed cases, including NHS number, patient first name, surname, date of birth, referring hospital, sample source (blood, CSF, etc.), meningococcal capsular group, molecular analysis and date of death (if died).
Office for National Statistics death registrations (ONS)	The Office for National Statistics (ONS) (www.statistics.gov.uk) provides PHE a record of annual death registrations in England for public health surveillance purposes and routinely converts the text within death certificates into ICD-10 codes. Data contained in the ONS database includes NHS number, patient first name, surname, date of birth, sex, place of residence, date of death, place of death and cause of death in free text and ICD-10 codes.
PHE LabBase2	PHE manages a central database (LabBase2) that collects routine electronic laboratory reports of clinically significant isolates reported voluntarily by NHS hospital laboratories in England. Data contained within LabBase2 include NHS number, patient first name, surname, date of birth, sex, reporting hospital, date of specimen, pathogen and antimicrobial susceptibility profile.
Private laboratory reports	Although MRU processes nearly all meningococcal isolates and clinical specimens for PCR-testing from NHS hospital laboratories in England, a few hospitals send some of their clinical specimens to one major private medical micro-pathology laboratory for PCR-testing. This laboratory routinely reports all PCR-positive IMD confirmations to PHE.
Hospital Episode Statistics (HES)	This database is managed by the Health and Social Care Information Centre and contains details of all admissions to NHS hospital trusts in England (~11 million episodes/year). HES contains a wide range of information, including NHS number, date of birth, sex, place of residence, ethnicity, admitting hospital, underlying medical conditions, timing and duration of inpatient-stay, reasons for admission and outcome at discharge. Potential episodes associated with a meningococcal infection were extracted by searching for any meningococcal (A39*), meningococcal-related (M010A, meningococcal arthritis; M030A; post-meningococcal arthritis) or infectious meningo-encephalitis (G00*) ICD-10 code in either the primary or the 19 secondary diagnostic codes. This is in contrast to published HES data that only report diagnoses in the primary diagnostic column. Data for linkage to MRU cases included NHS number, sex, DOB and postcode.

Prior to linkage, the Hospital Episode Statistics (HES) spells and episodes were converted to individual admissions. The electronic Patient Demographic Service (PDS) was used to maximise the number of cases with an NHS number using other identifiable data for individual cases. PDS is a national electronic database of NHS patient demographic details that allows healthcare staff to identify patients quickly and accurately (http://systems.hscic.gov.uk/demographics/pds). For all interrogated datasets, cases linked by NHS number were verified using the other linkage parameters (date of birth, sex, etc.) before being considered exact matches. The remaining unmatched cases where then matched using date of birth, gender and postcode, followed by different combinations of approximate date of birth, gender, region and approximate time of infection. All cases were then linked with the ONS death registration records to identify any extra cases not reported in the MRU or HES dataset, to ensure more complete outcomes for linked cases and to ascertain cause of death. Because LabBase2 and the private laboratory provided very few additional laboratory-confirmed cases to the total MRU-confirmed cases, these two data sources were interrogated at the end to identify any HES cases that might have been confirmed by laboratories other than the MRU. For LabBase2, individual clinical specimen records with an organism code for NEISSERIA MENINGITIDIS and with a specimen date in the study period were extracted, de-duplicated and linked to the MRU database. Linkage was done using NHS Number, patient surname, forename, date of birth, date of specimen, postcode and reporting laboratory. Any remaining episodes that did not link to the MRU database were then linked to the HES database. Case fatality ratio (CFR) refers to a fatal outcome within 30 days of a positive laboratory test and, where this date was not available, then the date of admission was used as a proxy. Patients with the same postcode for place of residence and place of death were considered to have died at home.

## Results

### MRU dataset

During 2007–11, the MRU confirmed 5115 IMD cases and 53,806 blood/CSF samples tested PCR-negative for IMD. Annual MRU-confirmed cases (and negative PCR-tests) were 1213 (10,112), 1170 (10,892), 963 (10,939), 885 (10,884) and 884 (10,979) for the 5-year period. Capsular group data were available for 4,963 cases and included MenB (*n* = 4435, 89 %), MenY (*n* = 278, 6 %), MenW (*n* = 124, 3 %), MenC (*n* = 115, 2 %) and other capsular groups (*n* = 11).

### HES dataset

During the same 5-year period, 62,183 total episodes with the selected ICD-10 codes for meningococcal disease (A39*) or any cause of bacterial meningo-encephalitis (G00*) were extracted, resulting in 61,867 episodes after de-duplication, equivalent to 42,558 admissions for 31,004 individuals. Of these, 8470 (20 %) admissions and 6727 (22 %) individuals had an A39* ICD10 code.

### Linking MRU cases to HES

#### MRU+/HESA39+ cases

**Fig 1:**
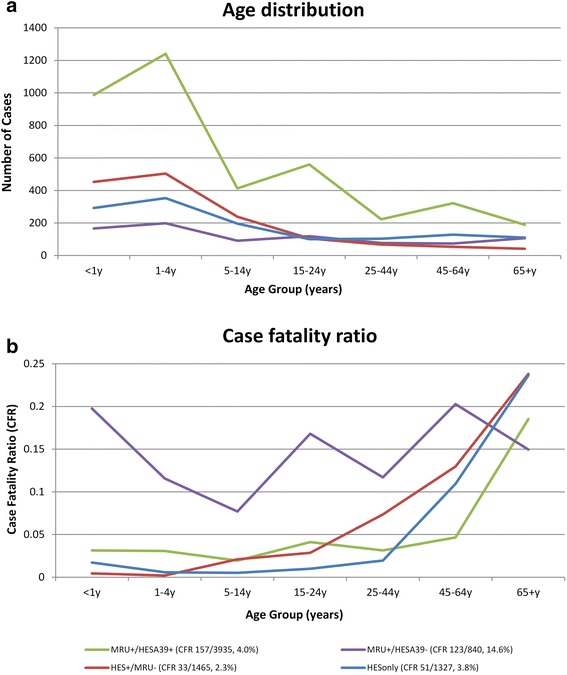
Age distribution of cases (**a**) and case fatality ratio (**b**) by age-group and linkage group in England during 2007–11

Of the 5115 MRU-confirmed cases, 4275 (84 %) matched exactly to a HES admission, including 3935 (92 %) matched to an A39* (meningococcal disease) diagnosis and 340 (8 %) to a G00* (bacterial meningo-encephalitis) diagnosis. The age distribution indicated a higher proportion of cases in young children with a peak among 1–4 year-olds and smaller peaks among 15–24 and 45–64 year-olds (Fig. 1a). Case fatality for this group was 4.0 % overall, with CFR remaining <5 % for all age-groups except ≥65 year-olds, where it was significantly higher (Fig. 1b).

#### MRU+/HESA39- cases (*n* = 840)

**Fig. 2:**
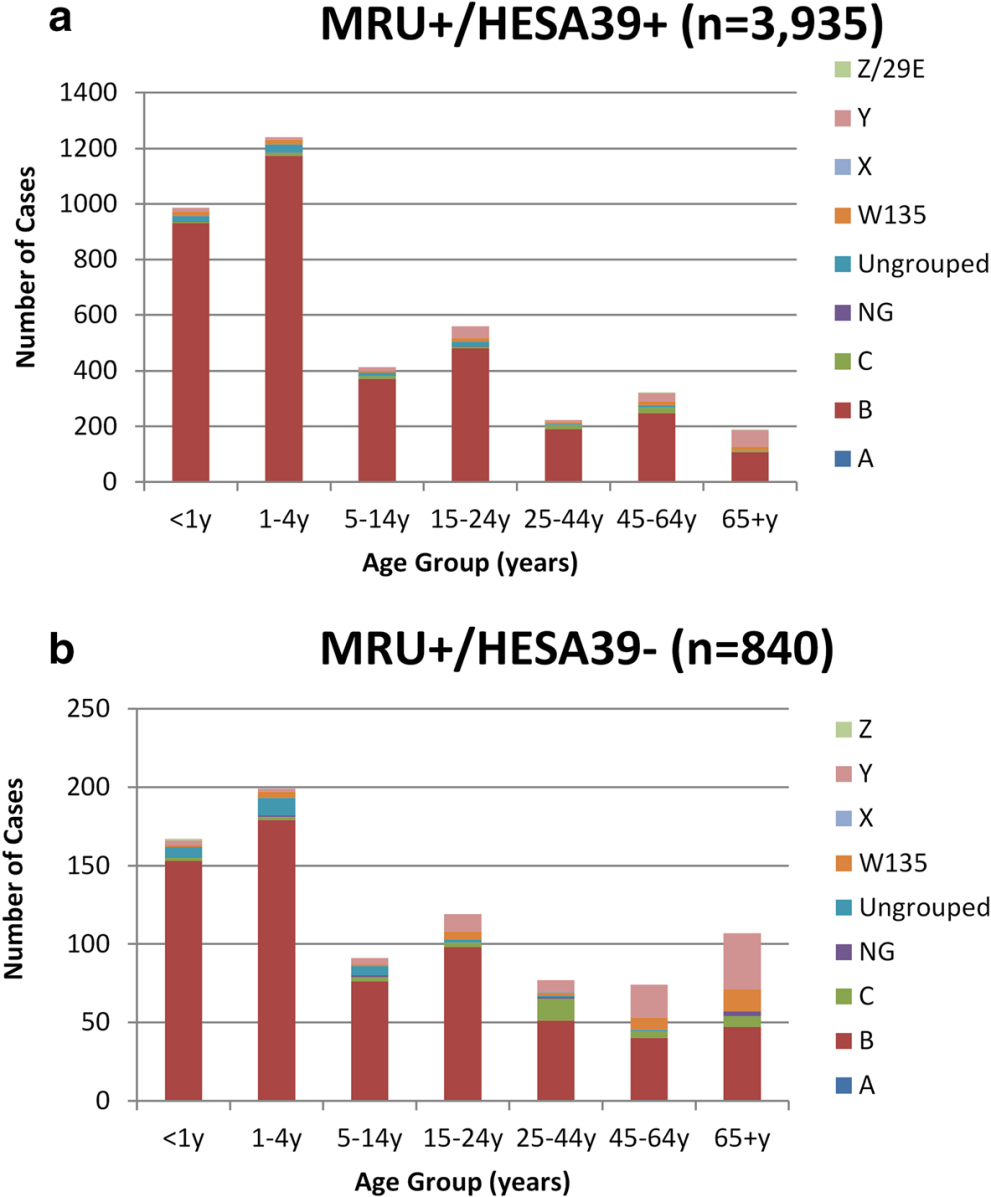
Comparison of age distribution between MRU-confirmed cases in England during 2007-11that linked with a HES A39* diagnosis (Fig. 2a) and those that did not (Fig. 2b). *Note the differing Y-axis between the two graphs*

Comparison of the 3935 MRU+/HESA39+ cases with the 840 MRU-confirmed cases that did not match to HES (i.e. MRU+/HESA39-) indicated that children and young adults aged <25 years (81 % vs. 69 %), and those with MenB disease (89 % vs. 77 %), were more likely to be recorded as IMD in HES (Fig. 2). In a logistic regression model with MRU+/HESA39+ and MRU+/HESA39- as outcome variables (0/1), failure to link with a HESA39* code was independently associated with increasing age in years (aOR 1.008, 95 % CI 1.004-1.011; *P* < 0.001), non-menB cases (2.0; 95 % CI, 1.6-2.5; *P* < 0.001) and fatal cases (aOR 3.9, 95 % CI 3.0-5.0; *P* < 0.001).

Upon further analysis of the 840 MRU+/HESA39- cases, 555 had an NHS number recorded. These cases were re-interrogated with the HES database to ascertain their discharge diagnoses. Of these, 456 had a HES admission between March 2006 and April 2012, including 345 (62 %) within 30 days of the MRU-positive test. Most MRU-confirmed cases could be linked to an infection-related HES admission around the time of sample receipt at the MRU (Table 2).

**Table 2:** Summary of the 840 IMD cases diagnosed in England during 2007–2011 that were confirmed by PHE MRU but could not be linked to a Hospital Episode Statistic (HES) admission matched to an A39* (meningococcal disease) or G00* (bacterial meningo-encephalitis) diagnosis (MRU+/HESA39- cases)

Outcome of linkage	Comment
Linked with a non-specific Infection-related code (*n* = 248)	These MRU-confirmed cases did not link with a HES A39* (meningococcal disease) or G00* (bacterial meningo-encephalitis) diagnosis but had non-specific infection-related codes, such as rash (*n* = 75), unspecified fever (*n* = 73), nausea and vomiting (*n* = 45), unspecified septicaemia (*n* = 31), unspecified viral infection (*n* = 23), lobar pneumonia (*n* = 17), unspecified pneumonia (*n* = 13) and unspecified viral meningitis (*n* = 12). Compared to children and adults, those aged ≥65 years were over-represented among the unlinked cases, possibly because IMD was less likely to be considered in the differential diagnosis for this age group. The ≥65 year-olds were also more likely to have a non-meningococcal cause (e.g. pneumococcal pneumonia) recorded in their discharge diagnosis
NHS numbers available but did not link to HES admission (*n* = 210)	These MRU-confirmed cases had NHS numbers but did not link to a HES admission within 30 days of sample receipt. They were more likely to be infants, toddlers or young adults (15–24 year-olds) and had the highest case fatality across the age groups (35 %) (Table 3). The most likely explanation for non-linkage is that these patients died before they could be hospitalised
Another pathogen was recorded in HES (*n* = 71)	These MRU-confirmed cases were coded in HES as having another infection, such as group B streptococcal (*n* = 14), *S. pneumoniae* or *E. coli* infection, or simply Gram-negative septicaemia (*n* = 12).
Cases without an infection-related code (*n* = 26)*	This was the smallest group where the MRU-confirmed case linked to a HES admission that did not have an infection-related code. *Two cases had a HES discharge diagnosis of “diagnosis not known”.* This could be coding error in HES or the patient may have been hospitalised with another illness within 30 days of MRU-confirmation of IMD
No NHS Number and not linked to HES admission (*n* = 285)	These cases followed a similar age-distribution as MRU-confirmed IMD cases, which may suggest that they are genuine IMD cases but could not be linked to HES because of lack of sufficient identifiers.

*IMD* invasive meningococcal disease, *MRU* Meningococcal Reference Unit, *HES* Hospital Episode Statistic, *NHS* National Health Service

Notably, the overall CFR for the MRU+/HESA39- cohort was higher than would be expected for IMD (14.5 %), especially among those with an NHS number recorded but did not link to a HES admission (NHS number+/Unlinked) (Table 3). Analysing the timing of MRU-confirmation of IMD and date of death revealed that nearly all confirmations within 30 days of death occurred on the day or in the days *after* the patient died (Additional file 1: Figure S1), suggesting that these patients had not been admitted to hospital and had died either in the Emergency Department or outside the hospital. This was generally true for all subgroups within the MRU+/HESA39- cohort and interrogation of the death registration records identified at least 19 patients who had died at home. Interestingly, although the HES ICD-10 codes for the MRU+/HESA39- cohort ranged between infection-related, another-pathogen-recorded, and non-infection codes, the death registration records for nearly all of the 122 fatal cases specifically documented IMD as the cause of death, with only six recording a non-specific infection-related death (Table 3).

**Table 3:** Number of deaths, cases and case fatality ratio (CFR, %) by age group for the 840 MRU-confirmed cases that did not link to a HES meningococcal disease ICD10 code (MRU+/HESA39-)

Age group	Linked+/Infection-related	NHS number+/Unlinked	Linked+/Another-pathogen	Linked+/Not-infection	No NHS number	All cases
<1y	5/50 (10.0 %)	20/43 (46.5 %)	0/9 (0.0 %)	1/2 (50.0 %)	7/63 (11.1 %)	33/167 (19.8 %)
1-4y	0/62 (0.0 %)	16/33 (48.5 %)	2/23 (8.7 %)	0/4 (0.0 %)	5/77 (6.5 %)	23/199 (11.6 %)
5-14y	0/33 (0.0 %)	5/16 (31.3 %)	0/5 (0.0 %)	0/3 (0.0 %)	2/34 (5.9 %)	7/91 (7.7 %)
15-24y	5/25 (20.0 %)	10/51 (19.6 %)	1/4 (25.0 %)	0/2 (0.0 %)	3/37 (8.1 %)	19/119 (16.0 %)
25-44y	1/11 (9.1 %)	6/25 (24.0 %)	0/2 (0.0 %)	0/2 (0.0 %)	2/37 (5.4 %)	9/77 (11.7 %)
45-64y	1/21 (4.8 %)	12/25 (48.0 %)	0/6 (0.0 %)	0/4 (0.0 %)	2/18 (11.1 %)	15/74 (20.3 %)
≥65y	5/46 (10.9 %)	5/17 (29.4 %)	3/22 (13.6 %)	2/9 (22.2 %)	1/13 (7.7 %)	16/107 (15.0 %)
Total	17/248 (6.9 %)	74/210 (35.2 %)	6/71 (8.5 %)	3/26 (11.5 %)	22/285 (7.7 %)^a^	122/840 (14.5 %)^a^
Cause of death on death registration record	Bacterial meningitis (2); Viral meningitis (1); Encephalitis (1); IMD (all others)	IMD (all fatalities)	Bacterial Meningitis (1); IMD (all others)	IMD (all fatalities)	Bacterial Meningitis (1); IMD (all others)	

The highest case fatality was observed for the MRU-confirmed cases with NHS numbers that did not link to a HES admission for meningococcal disease (NHS number+/Unlinked). Notably, the ONS death registrations recorded meningococcal disease as the cause of death for nearly all fatal cases irrespective of the linkage status among these MRU+/HESA39- cases IMD, Invasive meningococcal Disease; MRU, meningococcal reference unit; HES, Hospital Episode Statistic; NHS, National Health Service

^a^The age of six MRU-confirmed cases with no NHS number was not known

### HES cases that did not link to MRU-confirmed cases

**Fig. 3:**
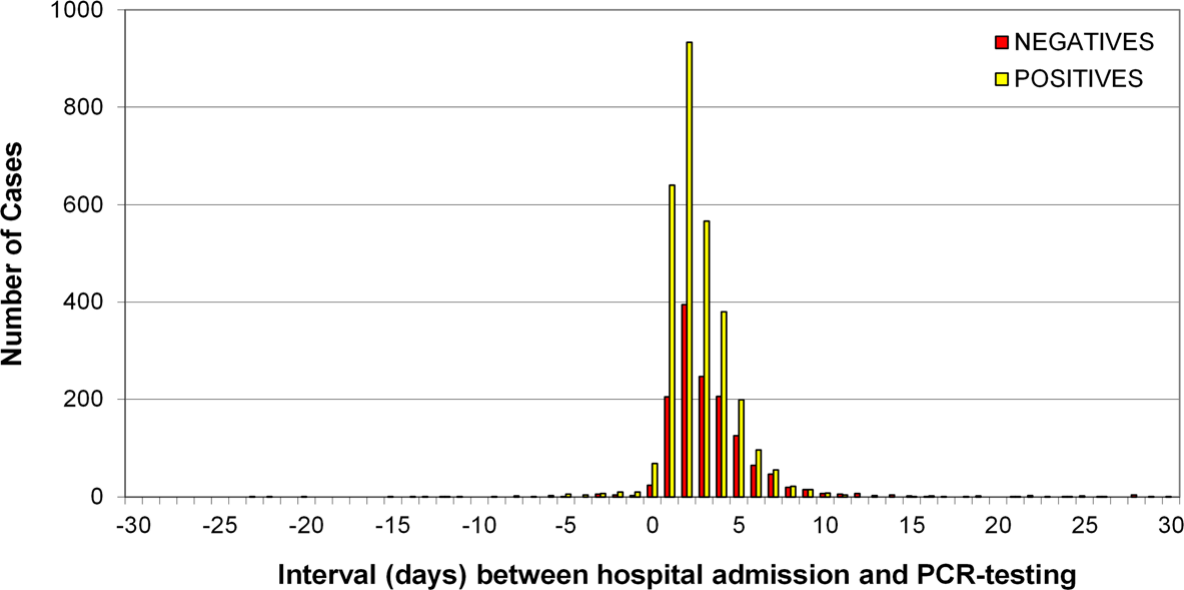
Interval in days between the hospital admission date and the PCR-testing date for MRU-confirmed cases that were coded as meningococcal disease (A39*) in HES (MRU+/HESA39+) and for HES cases coded as meningococcal disease (A39*) in HES but with a negative PCR-test by the MRU (HES+/MRU-)

Since only 3935 (58 %) of the 6727 HES cases with an A39* ICD-10 code (meningococcal disease) linked with the MRU-confirmed cases, there were, therefore, an additional 2792 (42 %) HES A39*cases that were not confirmed by the MRU. These additional cases were interrogated against 53,806 MRU PCR-negative reports and 1465 (52 %) matched to a PCR-negative result (HES+/MRU- cases). These cases, therefore, represent a cohort that had a diagnosis code for IMD in HES even though the patient had been tested by the MRU as PCR-negative. The interval between the date of hospital admission and the date when the PCR-test sample was received at the MRU was similar for MRU+/HESA39+ and HES+/MRU- cases (Fig. 3), suggesting that delay in PCR-testing from the date of admission was unlikely to explain the PCR-negative result.

Additionally, there were a further 1327 HESonly cases with an A39* ICD-10 code did not match to MRU-positive or MRU-negative cases. Interrogation of these cases with LabBase2 and private microbiology laboratory reports identified 43 and 30 additional cases that linked to the HESonly cases, respectively; these were, therefore, additional laboratory-confirmed IMD cases that had not been included among the MRU-confirmed cases. Assuming no duplication between these two additional data sources, this would suggest that the remaining (1327-43-30=) 1254 cases, equivalent to 251 annual cases, had no evidence of laboratory-confirmation for IMD, indicating that these cases were most likely diagnosed clinically . The age-distribution of HESonly cases (Fig. 1a) shows a predominance of cases in infants, toddlers and older children (the age groups considered to be at highest risk of IMD), with few cases in adults.

**Fig. 4:**
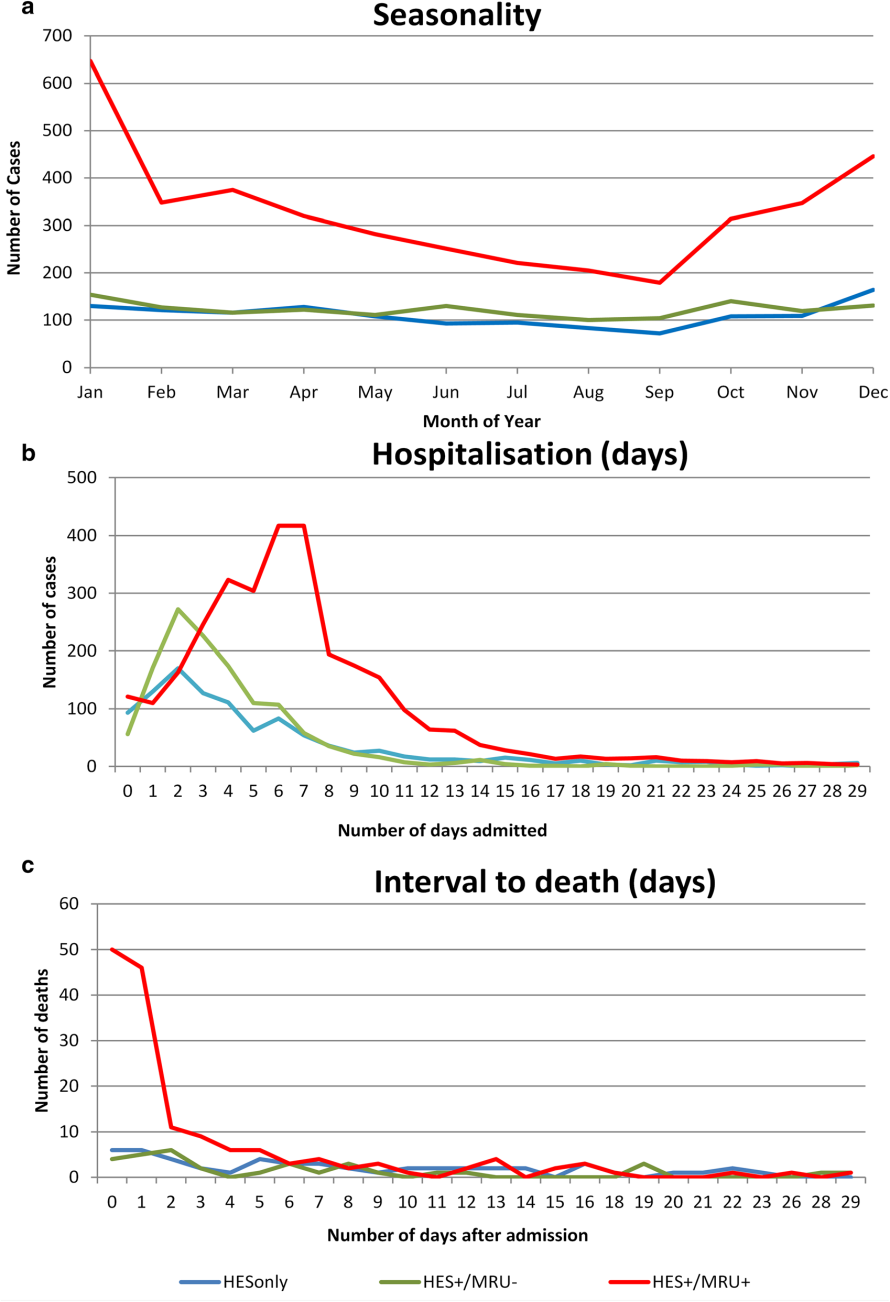
Seasonality (**a**), duration of hospital admission in days (**b**) and time from hospital admission to death in days (**c**) for clinically diagnosed HESonly cases without laboratory confirmation, HES cases with a negative MRU PCR-test (HES+/MRU-) and hospitalised, laboratory-confirmed IMD (MRU+/HESA39+) cases

Assessment of the seasonality (Fig. 4a) and duration of hospital admission for HESonly patients (Fig. 4b), showed a similar distribution to HES cases with a negative MRU PCR-test (HES+/MRU-) compared to hospitalised, laboratory-confirmed IMD (MRU+/HESA39+) cases. In particular, for cases with admission and discharge dates recorded, both the HESonly cases (393/1054, 37.3 %) and HES+/MRU- cases (499/1294, 38.6 %) had a higher proportion of patients admitted to hospital for ≤48 h compared with 12.9 % (394/3060) for MRU+/HESA39+ cases. Moreover, MRU+/HESA39+ cases (840/3935, 21.3 %) were significantly more likely to have multiple hospital admissions compared with HES+/MRU- (214/1465, 14.6 %) or HESonly (205/1327, 15.4 %) cases, and, in <5 year-olds, more likely to have multiple ICD-10 codes (in addition to A39*) recorded in HES (67.3 % vs. 57.3 % and 57.7 %, respectively, *P* < 0.0001), suggestive of more severe disease/complications.

Additionally, although CFR among HESonly cases was 3.8 % (51/1327 cases), the age distribution of CFR showed similarities to the HES+/MRU- cases, with extremely low CFR in children and then a rapid increase in CFR among older adults (Fig. 1b). Of these 51 deaths occurring within 30 days of admission among HESonly cases, 30 linked to a death registration record with free text information; of these, only 10 (33 %) recorded meningococcal infection as the cause of death; 12 (40 %) documented “meningo-encephalitis”, 6 (20 %) had another pathogen recorded, including GBS (*n* = 2), pneumococcal (n = 2) and streptococcal (*n* = 1) infections as well as staphylococcal meningitis with ventriculitis (*n* = 1); and two others (7 %) died of complications following a non-IMD infection. Moreover, analysis of the interval between hospital admission and death among fatal cases showed that MRU+/HESA39+ cases were significantly more likely to die within the first two days after hospital admission compared to the other two groups (Fig. 4c).

### LabBase2 cases

Of the 3278 invasive *Neisseria meningitidis* records in LabBase2, almost all (*n*=3149, (96 %) were also confirmed by the MRU. The remaining 129 were then matched against the HES dataset and 60 were subsequently linked, including 43 that linked to a HESonly case, six to HES+/MRU- and 11 to a HES G00-coded case. The remaining 69 cases (equivalent to 14 cases/year), therefore, did not link to HES or MRU cases.

### ONS death registrations

Interrogation of deaths registration data identified 3325 cases with an A39* code as a cause of death and/or “meningo-” and/or “encephalitis” in the free text. Of these, only 92 additional cases were identified that did not link to either the MRU-confirmed or HES IMD cases. Manual inspection of these records identified 34 non-meningococcal deaths (e.g. meningo-myelocele, or meningo-encephalitis cause by another pathogen), four non-infectious deaths in individuals who had recovered from a previous meningococcal infection, and two deaths with meningo-encephalitis and no pathogen recorded. Additionally, there were 14 deaths recorded with Waterhouse-Friderichsen Syndrome, including four associated with a non-meningococcal infection. Therefore, only 38 deaths were recorded as meningococcal disease over the 5-year period. Two of the 38 death records also mentioned Waterhouse-Friderichsen syndrome. There were, however, ten other death registration records that also noted Waterhouse-Friderichsen syndrome – these may or may not have been associated with IMD.

### Private laboratory reports

During 2007–11, PHE received 95 private microbiology laboratory reports of IMD confirmations. Of these, ten appeared to be cultures submitted as part of a nasopharyngeal carriage study and four others were non-invasive isolates. Of the remaining 81 cases, 71 (88 %) linked to the MRU/HES dataset, including MRU+/HESA39+ (35/71, 49 %) and HESonly (30/71, 42 %). Four additional reports tested PCR-negative at the MRU (HES+/MRU-), but were recorded as IMD in HES. Of these 4 reports, two PCR tests were performed more than a week after hospital admission and two were performed on the day after admission. Two additional results linked to MRU+/HESA39- cases. Assuming that the no further linkage of cases was possible, the private laboratory reports contributed to a maximum of 44 cases (equivalent to nine cases/year) in addition to the MRU-confirmed cases over the 5-year period (30 HESonly, 10 unlinked, 4 HES+/MRU-).

## Discussion

The linkage of multiple national data sources has provided useful insight into the total burden of IMD in England. The provision of free, national PCR-testing of clinical samples in addition to species confirmation and capsular grouping of clinical isolates by the MRU has not only guided clinical management of patients, but also improved case ascertainment and completeness of IMD surveillance [6]. LabBase2 and private laboratory reports added only a few more cases to the total number of confirmed IMD reports by the MRU, thus confirming two recent publications which reached the same conclusions [5, 6].

We successfully linked 4275 MRU-confirmed cases over the five-year period to a hospitalisation record in HES, where IMD (A39*) or bacterial meningo-encephalitis (G00*) was recorded. The remaining 840 (16 %) MRU-confirmed cases did not have sufficient identifiers for successful linkage, had non-specific infection-related ICD10 codes in HES, or died prior to hospital admission – either in the Emergency Department or outside hospital (e.g. at home).

At the same time, interrogating HES for IMD admissions identified an additional 2792 cases but more than half matched to an MRU PCR-negative test-result. Cross-checking these HES+/MRU- cases with LabBase2 and private laboratory reports identified only ten additional cases with positive laboratory confirmation. Discrepancies between the positive LabBase2/private laboratory testing and negative MRU PCR-tests may be due to the timing of sample submission or in the sensitivity of the diagnostic tests used. Overall, however, almost 1500 patients (equivalent to 300/year) had an IMD diagnosis in HES with a PCR-negative test by the MRU.

Of the remaining 1327 HES cases that did not match any MRU-tested samples, only 73 had additional laboratory-confirmation through LabBase2 or the private laboratory over the five-year period. This group also had the lowest proportion of identifiable information for linkage. It is possible that, if sufficient identifiers had been available, at least some of MRU-positive cases that did not link to HES might have linked to these HESonly cases. This would, however, still not account for >1000 HESonly cases (equivalent to >200/year) without any laboratory-confirmation for IMD.

The characteristics of the HESonly cases – age distribution, seasonality, short duration of in-patient stay (≤48 h in 40 %) and CFR – resembled HES+/MRU- cases more than MRU+/HESA39+ cases. In clinical practice, children (and many adults) often present with non-specific symptoms and signs such as fever and petechial rash and these cases usually end up being coded in HES as IMD because of lack of an alternative, more conclusive diagnosis. At the same time, however, some of these patients will have clinically-diagnosed IMD cases without laboratory-confirmation (e.g. those who received antibiotics prior to blood culturing and did not have a sample submitted for PCR-testing), although it is difficult to estimate the contribution of such cases to total HESonly cases.

In 1998, when the UK was experiencing a national MenC outbreak, we had estimated that there were 46 % more A39+ cases recorded in HES (*n* = 3316) than confirmed by the MRU (*n* = 2,276) [9]. This was a crude estimate of under-ascertainment because individual-level data-linkage was not performed. The current study has demonstrated the additional value of linking multiple national data sources whilst highlighting the complexities of linking large datasets that were not designed for integration. A straightforward comparison would indicate 32 % more HESA39+ (*n* = 6727) than MRU-confirmed (*n* = 5115) cases during 2007–11, which is lower than 46 % reported in 1998, possibly because of a higher proportion of cases with laboratory testing and confirmation [6]. Linkage of individual patient records, however, showed that a significant proportion of MRU-confirmed cases were not coded as IMD (A39+) in HES or died before admission to hospital. At the same time, a substantial proportion of hospitalised patients with a HES A39+ discharge code were either identified as PCR-negative by the MRU or were not laboratory-confirmed. During 2007–11, therefore, the latter group comprising 1254 HESonly cases would add 25 % more cases to the 5115 MRU-confirmed cases compared to our previous estimate of 46 % excess cases in 1998 [9].

Increasing use of NHS numbers for individual patients was critical in facilitating successful linkage, as did access to the patient demographic service (PDS) batch-testing which provided NHS numbers for cases with partial identifiers, highlighting the need for a unique identifier across all data sources for future linkage. For cases with missing NHS number, linkage success fell rapidly, making it difficult to ascertain whether the additional unlinked records are genuinely more cases or simply unlinked duplicates. Despite the wealth of data generated, it is clear that linkage of national datasets in their current format is labour-intensive and not amenable to automation, but could be repeated at regular intervals to monitor trends over time.

## Conclusions

Our analysis confirms that the MRU captures nearly all laboratory-confirmed IMD cases and is, therefore, ideal for monitoring national trends in near-real time. Linkage with HES, however, identified an additional 2792 hospitalised cases over 5 years with an IMD diagnostic code, including half that linked to an MRU PCR-negative result; of the remaining cases, it is difficult to estimate what proportion were genuine but unconfirmed IMD cases. Reassuringly, though, these additional HES cases indicate increased awareness of IMD among clinicians with low thresholds for hospitalising patients with suspected IMD. Following the introduction of a broad-spectrum meningococcal vaccine (4CMenB; Bexsero, GSK Biologicals) into the UK childhood immunisation programme in September 2015, monitoring trends in laboratory-confirmed and unconfirmed hospitalised IMD cases could provide useful insight into the true burden of IMD. In the meantime, our results justify the continued use of MRU data for monitoring IMD epidemiology in England.

## Additional file

Additional file 1: Figure S1. Interval between PCR-testing by the Meningococcal Reference Unit (MRU) and death for the different subgroups within the cohort of 840 cases that were MRU-confirmed but did not link to a hospital admission with an A39* (meningococcal disease) or G00* (bacterial meningo-encephalitis) diagnosis code (MRU+/HESA39- cases). Nearly all samples for all subgroups were tested on or after the day of death (i.e. negative interval), suggesting that the patient died without being admitted to hospital, for example, in the Hospital Emergency Department or at home. (TIFF 299 kb)

## Abbreviations

DOB: Date of birth
aOR: Adjusted Odds Ratio
CFR: Case Fatality Ratio
HES: Hospital Episode Statistics
IMD: Invasive Meningococcal Disease
MRU: The Public Health England Meningococcal Reference Unit
NHS: National Health Service
ONS: Office for National Statistics
PCR: Polymerase Chain Reaction
PDS: Patient Demographic Service
PHE: Public Health England
