# Supplementary figures and images for: Invasive meningococcal disease in England: assessing disease burden through linkage of multiple national data sources
Source: BMC Infect Dis. 2015 Dec 1;15:551. doi: 10.1186/s12879-015-1247-7 (PMC4667514; doi:10.1186/s12879-015-1247-7)

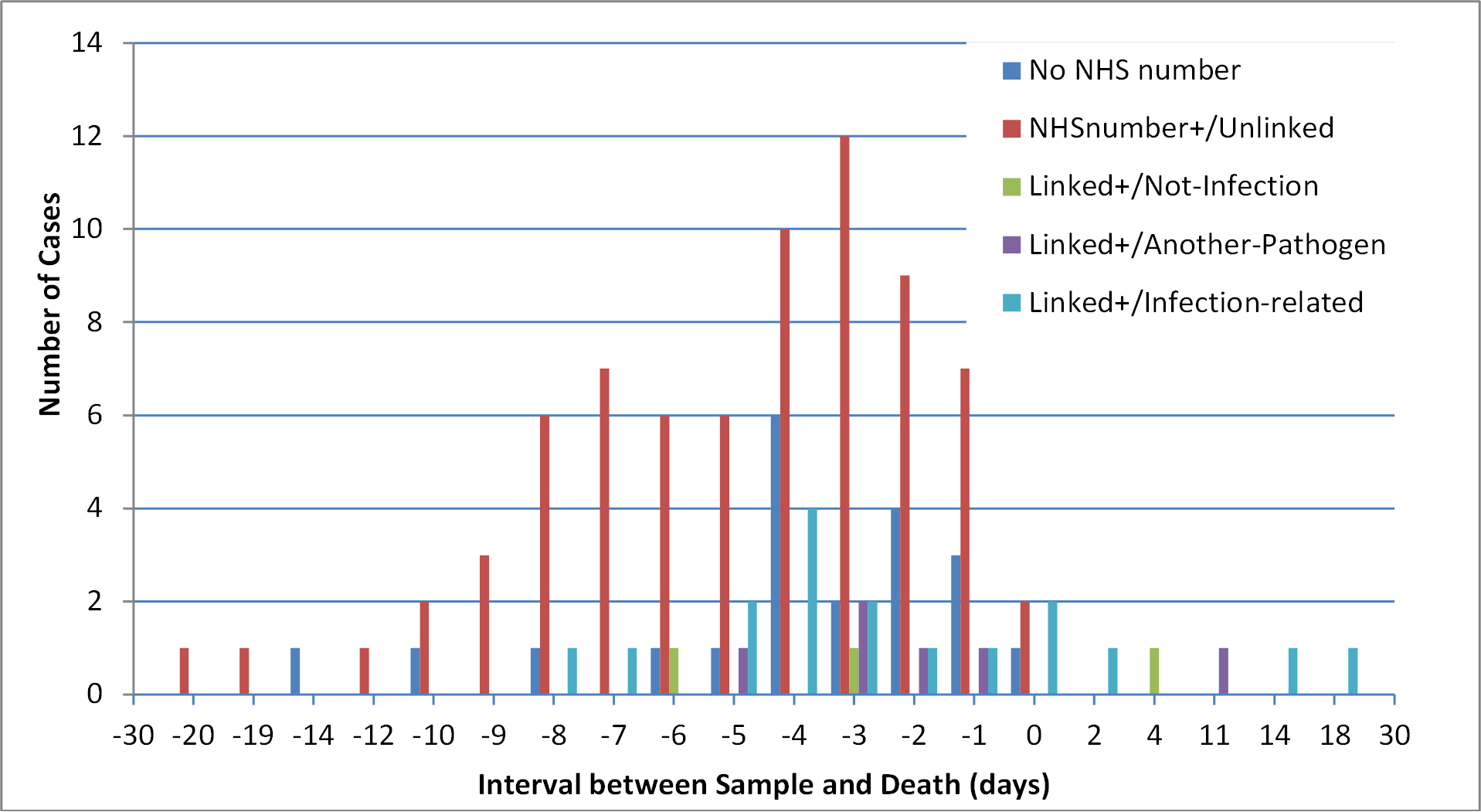

Supplement: Additional file 1: Figure S1. — Interval between PCR-testing by the Meningococcal Reference Unit (MRU) and death for the different subgroups within the cohort of 840 cases that were MRU-confirmed but did not link to a hospital admission with an A39* (meningococcal disease) or G00* (bacterial meningo-encephalitis) diagnosis code (MRU+/HESA39- cases). Nearly all samples for all subgroups were tested on or after the day of death (i.e. negative interval), suggesting that the patient died without being admitted to hospital, for example, in the Hospital Emergency Department or at home. (TIFF 299 kb) [file 12879_2015_1247_MOESM1_ESM.tiff]
